# Preschoolers’ Beliefs, Emotions and Intended Responses toward Peer Behaviors: Do Children’s Sex, Age, and Social Behavior Make a Difference?

**DOI:** 10.3390/children10081312

**Published:** 2023-07-29

**Authors:** Maryse Guedes, Manuela Verissimo, António J. Santos

**Affiliations:** William James Center for Research, ISPA—Instituto Universitário, 1149-041 Lisbon, Portugal

**Keywords:** beliefs, intended affiliative preferences, child sex, child age group, child social behavior, early childhood

## Abstract

Children’s self-imposed isolation in the company of others (social withdrawal due to shyness or unsociability) and active isolation due to direct aggressive behaviors can challenge successful engagement in the peer group. The developmental attributional perspective acknowledges that children’s intended affiliative responses are, in part, guided by their emotions and beliefs toward peers’ social behaviors and may differ depending on children’s sex, age group, and social behavior. This study aimed to (1) describe preschoolers’ beliefs, emotions, and intended affiliative preferences toward aggressive, shy, and unsociable peers, depending on children’s sex and age group, and (2) explore the associations between preschoolers’ beliefs, emotions and intended affiliative preferences, depending on children’s social behaviors and children’s sex or age group. A total of 158 preschoolers aged 3–6 years were interviewed, using the Child Attributions Interview to assess their beliefs, emotions and intended affiliative preferences toward peers. Teachers completed the Social Competence and Behavior Evaluation Scale to assess children’s prosocial behaviors, aggressive-anger, and anxious-withdrawal. Preschoolers’ perspectives toward shy and unsociable peers were less negative than toward aggressive peers. However, participants in our sample were not fully aware of the different intentionality and social motivations of shy and unsociable peers. Higher levels of perceived social standing, social motivation and sympathy predicted higher affiliative preferences toward shy, unsociable, and aggressive peers. However, the magnitude of the associations between preschoolers’ beliefs, emotions and intended affiliative preferences differed, depending on children’s sex, age group and prosocial behavior, especially toward aggressive and shy peer behaviors. These findings are in line with the developmental attributional perspective, but highlight the need to account for developmental specificities, sex differences in peer relationships and children’s individual differences in social behaviors.

## 1. Introduction

Positive involvement in the peer group is a key developmental issue during the preschool years [1]. Children’s self-imposed isolation in the company of others (social withdrawal) and children’s active isolation by peers due to deviations from normatively acceptable behaviors can challenge their successful involvement in the peer group [2].

Contemporary scholars distinguish different subtypes of socially withdrawn behaviors that encompass distinct emotional, motivational, and psychological substrates [3]. Shyness refers to a temperamental trait that manifests itself through wariness in unfamiliar social situations and self-consciousness in situations of perceived social evaluation [2]. From a motivational standpoint, scholars consider that shy preschoolers may experience a desire of social approach that coexists with feelings of social anxiety, displaying reticent or onlooking behaviors during play activities in the classroom [4]. Conversely, unsociability is defined as a non-fearful preference for solitude [2] that reflect a reduced desire for social approach without actively avoiding social situations [4]. Unsociable children are able to engage in socially competent behaviors when approached by peers [5]. Shyness and unsociability distinguish themselves from children’s active isolation by peers [2,6], due to frequent direct aggressive behaviors that encompass intended acts to hurt and harm peers (e.g., pushing, or harmful communication) during face-to-face interactions [7].

Higher levels of shyness [8,9] and direct aggression [10] during early childhood have been concurrently and longitudinally associated with lower peer acceptance and negative peer experiences. Although relatively benign during early childhood, preliminary evidence suggests that peers might begin to respond more negatively to unsociable children in middle and late childhood [11]. According to a developmental attributional framework [12], children’s intended affiliative responses toward peers’ social behaviors are, in part, guided by their emotions and beliefs about others’ thoughts and intentions. Understanding children’s beliefs, emotions and intended responses toward shy, unsociable, and aggressive behaviors is crucial to guide the design of evidence-based interventions that can enhance adaptive socioemotional outcomes.

### 1.1. Preschoolers’ Beliefs, Emotions and Intended Affiliative Preferences toward Peer Behaviors

Positive involvement in the peer group is, in part, associated with one aspect of social understanding that encompasses children’s ability to make inferences about others’ internal mental states, such as thoughts, emotions and desires [13]. Although children display a nascent ability to understand others’ desires and emotions in everyday social interactions during infancy, children’s beliefs, that is, children’s representations about others’ internal mental states develop significantly between ages 3 and 5 [14]. During this period, children begin to understand the notion that different people hold different representations of the social world that guide their behaviors [14]. Early research on children’s beliefs toward social withdrawal and direct aggression suggested that children aged 5 to 6 years hold less well-developed representations toward the former than the latter [15,16]. However, subsequent studies asking kindergarten and first-grade children (mean age: 6 years) from North American [12,17,18], Dutch [19] and Chinese [20,21] contexts to respond to hypothetical vignettes evidenced that children hold more sophisticated implicit representations about aggressive, socially withdrawn and socially competent peers.

More specifically, these studies found that hypothetical socially withdrawn peers were less likely to be described as desirable playmates and more likely to be perceived negatively than socially competent peers in terms of their social motivation and social standing in the classroom [17,20,21]. Nevertheless, kindergarten and first-grade children expressed higher sympathy [19] and affiliative preference [17] toward hypothetical socially withdrawn peers and considered their behaviors as less intentional [12] and less problematic in the classroom [17,18] than those of aggressive peers. Research showed that kindergarten and first-grade children appear to be also able to make fine-grained distinctions between shy and unsociable behaviors [17,20,21]. Children reported a greater affiliative preference toward hypothetical shy peers and considered that they were less intentional and more socially motivated, had a higher social standing and lower negative impact in the classroom, and elicited more sympathy than hypothetical unsociable peers [17,20,21].

More recently, studies have been conducted in samples of younger preschoolers (mean ages: 4 to 5 years) from Italian [22] and Argentinian contexts [23], using similar methods. In line with prior research involving elementary school children [12,17,19], preschoolers reported less negative views, more sympathy, and higher affiliative preference toward hypothetical socially withdrawn behaviors when compared with aggressive behaviors [22,23]. These studies also supported the idea that young children were able to distinguish hypothetical socially competent, shy, and unsociable behaviors in terms of their intentionality and social motivations [22,23]. However, preschoolers reported comparable social standing, negative impact, and affiliative preferences toward shy and unsociable peers [22,23]. Whereas [23] found no significant differences in sympathy for shy and unsociable behaviors, [22] showed that preschoolers reported more sympathy toward shy peers when compared with unsociable peers.

### 1.2. Associations between Preschoolers’ Beliefs, Emotions, and Intended Responses toward Peer Behaviors

To date, most research drawn on a developmental attributional framework [12] has explored the associations of children’s attributions of intentionality and sympathy with affiliative preferences toward peer behaviors [20]. Higher attributions of responsibility (e.g., [24,25,26,27]) and emotions of sympathy [12,28] toward hypothetical aggressive, socially withdrawn, clinically depressed and hyperactive peers were associated with lower intended affiliative preferences in samples of kindergarten and primary school children. A lower proportion of studies have focused on other types of children’s interpretations toward peer behaviors. These studies found that preschoolers who perceived the behaviors of aggressive and socially withdrawn peers as more controllable and stable reported lower intended affiliative preferences toward them (e.g., [12,29,30]). Unsociable and shy behaviors are thought to differ in terms of their intentionality, social motivation [3], negative impact, social standing in the peer group and have been associated with different peer experiences during early childhood [8,9,11]. Further studies are needed to explore the associations between children’s interpretations, emotions of sympathy and intended affiliative preferences toward shy and unsociable behaviors during the preschool years.

### 1.3. The Role of Children’s Sex, Age, and Social Behavior

Literature suggests that children’s sex needs to be considered, when examining the associations of children’s beliefs, emotions and intended affiliative preferences toward different peer behaviors. In fact, gender stereotypes of male dominance may influence how children perceive and react to distinct peer behaviors [31]. Consistent with this idea, research drawn on hypothetical vignettes’ methods found that boys displayed less negative views toward aggressive peers and perceived them as more desirable playmates than girls but expressed significantly less sympathy toward them than girls [17,19]. Shy and unsociable peers were considered less problematic [20], less intentional and more desirable playmates among girls [22].

Research also appears to support the idea that children’s ability to understand others’ thoughts and intentions may become more sophisticated with age. In fact, older children appear to be more able to distinguish aggressive and socially withdrawn children along a responsibility dimension [12] and to make more accurate inferences about the social motivations underlying peer behaviors, especially socially competent and unsociable ones [21]. Whereas [12] found that older preschoolers reported higher sympathy toward aggressive peers than younger preschoolers, [22] found that the intended affiliative preferences toward all hypothetical peers (except the aggressive one) were higher among older children.

Few studies also found that children’s own social behaviors may impact their beliefs, emotions, and responses toward peer behaviors [17,18]. More specifically, children who displayed higher levels of maternal-reported prosocial behaviors made more accurate inferences about the intentionality of shy peers but described higher sympathy and affiliative preferences toward the socially competent peer than less empathic and prosocial children [18]. In a subsequent study, [17] found that children rated as aggressive and unsociable by mothers reported significantly lower affiliative preferences toward all the hypothetical peers. However, aggressive children perceived that all hypothetical peers would cause more problems in the classroom, whereas unsociable children perceived hypothetical peers as less likely to be disruptive in the classroom [17].

Although parents are familiar with children’s cues in multiple contexts, preschool teachers observe children in daily peer interactions and develop standards of competent behaviors, based on their observation of many children of similar age [32]. Furthermore, studies found higher levels of prosocial behaviors and lower levels of aggression among girls when compared with boys and an increase in prosocial behaviors as children grow up [32,33,34,35,36]. Understanding the role of children’s social behaviors in the associations between beliefs, emotions, and affiliative preferences toward peer behaviors may be capitalized, by considering preschool teachers’ ratings and their addictive effects with children’s sex or age group.

### 1.4. The Present Study

To advance the current state-of-art knowledge, the present study aimed to (1) describe preschoolers’ beliefs, emotions and intended affiliative preferences toward aggressive, shy, and unsociable peers, depending on children’s sex and age group, and (2) explore the associations between preschoolers’ beliefs, emotions and intended affiliative preferences, depending on children’s social behaviors, children’s sex, and age group. This is the first study to examine preschoolers’ beliefs, emotions and intended affiliative preferences toward different peer behaviors in the Portuguese context. According to a bioecological developmental framework [37], culture may shape the beliefs, emotional and behavioral reactions toward children’s social behaviors in the peer group [38]. In Portugal, desirable qualities to be fostered in children continue to reflect both collectivist (i.e., good manners, responsibility, tolerance, and respect) and individualistic (i.e., independence and determination) values [39]. In line with other studies conducted in the Western context, children’s lower social competence and social engagement (e.g., [40,41,42]), less positive, more disrupted, and disconnected behaviors during peer play (e.g., [43]) were found to be associated with lower peer acceptance in samples of Portuguese preschoolers.

For the first aim, we hypothesize that preschoolers will display less positive views (i.e., social motivation, social standing, and negative impact) and lower affiliative preferences toward shy and unsociable hypothetical peers when compared with socially competent peers (H1). However, preschoolers are expected to display more positive views, higher sympathy, and affiliative preferences toward shy and unsociable hypothetical peers when compared with aggressive peers (H2). Furthermore, we hypothesize preschoolers will report comparable social standing, negative impact and affiliative preferences toward shy and unsociable behaviors but will distinguish them in terms of their intentionality and social motivations (H3). Due to the inconsistency of the findings in samples of younger preschoolers, we didn’t establish hypotheses for sympathy. With respect to sex differences, we expect that boys will display more positive views toward aggressive behaviors when compared with girls, whereas an inverse pattern is expected for shy, unsociable, and socially competent behaviors (H4). Furthermore, older (5–6 years) children are expected to display more accurate views toward peers’ intentionality and social motivation and higher affiliative preferences toward all peer behaviors (except aggression) when compared with younger (3–4 years) children (H5).

For the second aim, we hypothesize that preschoolers who will perceive higher levels of intentionality, lower social motivation and social standing, higher negative impact toward shy, unsociable, and aggressive peer behaviors and who will report lower sympathy will report lower affiliative preferences (H6). Due to the exploratory nature of available evidence, we didn’t establish hypotheses for the role of children’s sex, age group and social behaviors in the associations between preschoolers’ beliefs, sympathy and intended affiliative preferences.

## 2. Materials and Methods

### 2.1. Participants

Participants were 158 preschool children with a mean age of 57 months (*SD* = 11. 66) and 61% boys (*n* = 94) who were attending six private urban and semiurban preschools, from diverse socioeconomic backgrounds. Children’s preschool teachers (*n* = 22) had a mean age of 39 years (*SD* = 8.62), were mostly female (*n* = 20, 91%) and had a mean of 15 years (*SD* = 8.40) of professional experience.

### 2.2. Procedure

This study was approved by the ISPA Ethics Committee. The study objectives and procedures were presented to preschool boards. After approval by the preschool boards, parents/legal guardians of all children were asked to sign a written informed consent to authorize children’s and preschool teacher’s participation in the study. The acceptance rate for preschoolers’ participation was approximately 50%. Preschool teachers were also asked to provide their written informed consent. All the contacted preschool teachers (*n* = 23) agreed to participate.

Data collection procedures took place between February and June 2022. Before data collection, children were informed about the aims and procedures of the study, the voluntary nature of their participation and the confidentiality of their responses. Children’s verbal assent to participate was requested before starting the interview. The refusal of the minor to provide assent to participate was respected. After ethical procedures, a trained research assistant interviewed each child individually, in a private room at preschool. Each interview lasted about 15 min and was scheduled to minimize the interference of data collection in children’s daily routines and activities. Preschool teachers received a link to complete the questionnaires in an online protected platform (Qualtrics) by e-mail or a paper version of the measures in a closed envelope, according to their best convenience.

### 2.3. Measures

Child Attributions Interview: The Child Attributions Interview assesses children’s self-reported insights and liking of hypothetical peers, distinguishing different types of social behaviors (i.e., aggressive, shy, unsociable, or socially competent). This interview was drawn from the Interview for Attributions for Aggressive and Withdrawn Behaviors, originally developed to evaluate children’s beliefs, emotions and intended responses toward hypothetical aggressive and socially withdrawn peers [12] and later adapted to include a hypothetical socially competent [19], shy and unsociable peers [17]. Results from several previous studies provided strong evidence on the comprehensibility of the interview for young children and on its reliability and validity (e.g., [12,17,19,20,21,22,23]). The pictorial materials and the limited verbal response format used in the Child Attributions Interview have been found to be effective ways to obtain reliable and valid reports of internal constructs among young children [44]. The Portuguese version of the interview was established, using back-forward translation procedures. During the interview, the research assistant read four hypothetical vignettes, illustrating different social behaviors of same-sex peers (aggressive, shy-anxious, unsociable, and socially competent behaviors) in the classroom. Each vignette was accompanied by a cartoon picture. The presentation order of the vignettes was modified, using a Latin square design. After being read each vignette, children were asked seven questions, responded in a three-point scale (1—No/Never, 2—Maybe/Sometimes, and 3—Yes/A lot) by pointing circles of increasing sizes. Questions assessed the perceived intentionality (do you think that he/she act on purpose? do you think he/she want to behave like this?), social motivation (does he/she want to play with other kids?), social standing (would other kids in your class want to play with him/her?), negative impact (do kids that act like he/she cause a problem in your class?), sympathy (how much do you feel sorry for him/her?) and intended affiliative preference (would you like to play with him/her?, would you want to be his/her friend?) toward each of the presented hypothetical peers. Children’s responses to questions assessing perceived intentionality and affiliative preferences were combined to create an aggregate score for each of the hypothetical peer behaviors [17].

Social Competence and Behavior Evaluation Scale (SCBE-30) [32,34]: The SCBE-30 has been widely used in different cultures and provides a standardized description of affect and behavior in context, discriminating behavioral-emotional problems and social adjustment [34,35]. This rating scale, composed of 30 items, assesses preschool teachers’ perceptions about the overall quality of child’s adaptation during peer play, in terms of three dimensions: (1) Prosocial Behavior (10 items), designed to assess a well-adjusted, flexible, emotionally mature and generally prosocial pattern of prosocial adaptation; (2) Anger-Aggression (10 items), referring to a maladaptive pattern related to externalizing behaviors, including aggressive, selfish and oppositional behaviors; and (3) Anxiety-Withdrawal (10 items), measuring a maladaptive pattern related to internalizing behaviors, including anxious, depressed, isolated and overly dependent behaviors. Preschool teachers are asked to answer each item, using a 6-point Likert scale ranging from 1 (Never Occur) to 6 (Always). In the present sample, Cronbach’s alfas were 0.74 for Anxiety-Withdrawal and 0.88 for Prosocial Behavior and Anger-Aggression.

### 2.4. Data Analysis

For the first study aim, mixed repeated-measures ANOVAs were performed, using Child Sex (boys vs. girls) and Age Group (3–4 vs. 5–6 years) as between-subject factors and the type of peer behavior (aggressive, shy-anxious, unsociable, and socially competent peer behaviors) as a within-subject factor (since all children responded to the four vignettes depicting these kind of social behaviors). When a significant main effect of Type of Behavior or interaction effects were found, post-hoc paired *t*-tests comparisons with Bonferonni corrections were conducted.

For the second study aim, preliminary factorial ANOVAs, using Child Sex and Age Group as between-subject factors, were conducted to compare the teacher-rated social behaviors (i.e., prosocial behavior, aggression, and anxious withdrawal) of boys and girls aged 3–4 years and 5–6 years. Preliminary Pearson correlations analyses were conducted to examine the associations between preschoolers’ beliefs (i.e., perceived intentionality, social motivation, negative impact, and social standing), emotions (i.e., sympathy), and their intended affiliative preferences toward each of the presented problematic peer behaviors (i.e., shyness, unsociability, and direct aggression). Pearson and point-biserial correlation analyses were computed to identify potential sociodemographic covariates. Multiple linear moderated regression analyses were used to examine the previously identified significant associations between preschoolers’ beliefs, emotions, and intended affiliative preferences, depending on each children’s social behavior (i.e., prosocial behavior, aggression, or anxious withdrawal) and children’s sex and age group. These analyses were conducted in accordance with the recommendations of [45]. In the first step, each independent variable (i.e., perceived social motivation, social standing, or sympathy) and the moderators (i.e., sex, age group, teacher-rated prosocial behavior, aggression, or anxious withdrawal) were introduced. Continuous variables were mean-centered and dichotomous variables were dummy-coded (children’s sex: 1—male, 0—female; age group: 1–3–4 years, 0–5–6 years). In the second step, the interaction terms were introduced. When significant interaction effects were identified, simple slopes analyses were performed, using PROCESS model 1 (when a moderation effect was detected) or 2 (when more than a moderation effect was found to allow the effect of the independent variable on the dependent variable to vary with a moderator when a second one is held fixed) [46].

Statistical significance was set at *p* < 0.05, but marginally significant interactions (*p* < 0.10) were also reported. When significant interactions were identified, simple slopes calculations were reported and interpreted. Post-hoc power calculations with a significance level of 0.05 and power ≥ 0.80, using G-Power [47,48], showed that small effects could be detected (*f* = 0.09) for the first aim and small to medium effects (*f* = 0.07) could be detected for the second aim.

## 3. Results

### 3.1. Preschoolers’ Beliefs and Intended Responses toward Aggressive, Shy, Unsociable, and Socially Competent Peers, According to Children’s Sex and Age Group

#### 3.1.1. Perceived Intentionality

Table 1 shows that a significant main effect of Type of Social Behavior was found. Post-hoc comparisons with Bonferonni corrections showed that socially competent peer behaviors were considered as more intentional than aggressive (*t* = 4.61, *p* < 0.001, *d* = 1.04), shy (*t* = 6.83, *p* < 0.001, *d* = 0.54) and unsociable (*t* = 4.54, *p* < 0.001, *d* = 0.36) peer behaviors. A significant interaction between sex and age group was also found. In the 3–4 years age group, boys perceived unsociable (*t* = 2.49, *p* = 0.007, *d* = 0.56), aggressive (*t* = 2.26, *p* = 0.026, *d* = 0.52), socially competent (*t* = 2.03, *p* = 0.046, *d* = 0.46) and shy (*t* = 2.00, *p* = 0.048, *d* = 0.46) behaviors as more intentional than girls. In the 5–6 years age group, girls perceived unsociable behaviors as more intentional than boys (*t* = −2.54, *p* = 0.013, *d* = 0.59).

#### 3.1.2. Perceived Social Motivation

As shown in Table 1, a significant main effect of Type of Social Behavior was found. Post-hoc comparisons with Bonferonni corrections showed that aggressive peer behaviors as less socially motivated than unsociable (*t* = −2.83, *p* = 0.005, *d* = 0.23) and socially competent (*t* = −6.29, *p* < 0.001, *d* = 0.50) peer behaviors. Furthermore, socially competent peer behaviors were perceived as more socially motivated than shy (*t* = 4.02, *p* < 0.001, *d* = 0.32) and unsociable (*t* = 4.16, *p* < 0.001, *d* = 0.33) behaviors.

A significant main effect of Child Age Group was also identified. Children in the 3–4 years age group globally perceived all hypothetical peers as less socially motivated than children in the 5–6 years age group.

#### 3.1.3. Perceived Social Standing

Table 1 shows that a significant main effect of Type of Social Behavior was found. Post hoc comparisons with Bonferonni corrections showed that aggressive peers were perceived as less appreciated in the peer group than shy (*t* = −6.07, *p* < 0.001, *d* = 0.49), unsociable (*t* = −7.05, *p* < 0.001, *d* = 0.57), and socially competent (*t*= −6.69, *p* < 0.001, *d* = 0.70) peers.

Furthermore, shy peers were perceived as less appreciated in the peer group than socially competent peers (*t* = −2.73, *p* = 0.007, *d* = 0.22). A significant interaction between the Type of Behavior and Age Group was also found. The magnitude of the differences between aggressive and shy peers concerning perceived social standing was stronger in the 5–6 years age group (*t* = −6.08, *p* < 0.001, *d* = 0.70) than in the 3–4 years age group (*t* = −2.72, *p* = 0.008, *d* = 0.31).

#### 3.1.4. Perceived Negative Impact

As shown in Table 1, a significant main effect of Type of Social Behavior was found. Post hoc comparisons with Bonferonni corrections showed that aggressive peer behaviors were perceived as having a more negative impact than shy (*t* = 4.97, *p* < 0.001, *d* = 0.40), unsociable (*t* = 3.64, *p* < 0.001, *d* = 0.29), and socially competent (*t* = 6.10, *p* < 0.001, *d* = 0.49) peer behaviors.

#### 3.1.5. Sympathy

Table 1 shows that a significant main effect of Type of Social Behavior was found. Post hoc comparisons with Bonferonni corrections showed that aggressive peer behaviors elicited less sympathy than shy (*t* = −4.80, *p* < 0.001, *d* = 0.48), unsociable (*t* = −6.39, *p* < 0.001, *d* = 0.60), and socially competent (*t* = −4.77, *p* < 0.001, *d* = 0.46) peer behaviors.

#### 3.1.6. Intended Affiliative Preferences

Table 1 shows that a significant main effect of Type of Social Behavior was found. Post hoc comparisons with Bonferonni corrections showed that preschoolers reported a lower affiliative preference toward aggressive peers when compared with shy (*t* = −9.98, *p* < 0.001, *d* = 0.79), unsociable (*t* = −9.93, *p* < 0.001, *d* = 0.79), and socially competent (*t* = −10.71, *p* < 0.001, *d* = 0.85) peers. 

A significant interaction between the Type of Behavior and Age Group was also found. The magnitude of the differences in intended affiliative preferences toward aggressive peers when compared with shy (*t* = −8.49, *p* < 0.001, *d* = 0.97), unsociable (*t* = −8.54, *p* < 0.001, *d* = 0.98) and socially competent (*t* = −9.35, *p* < 0.001, *d* = 1.07) peers were stronger in the 5–6 years age group than in the 3–4 years age group.

### 3.2. Associations between Preschoolers’ Beliefs, Emotions, and Intended Affiliative Preferences, Depending on Children’s Social Behavior, Sex and Age Group

Table 2 displays the descriptive statistics (means and standard deviations) of teacher-rated Prosocial Behavior, Anger-Aggression and Anxious-Withdrawal, depending on children’s sex and age group. ANOVAs show that teacher-rated Prosocial Behavior was lower for boys than for girls and among children aged 3–4 years than among children aged 5–6 years. No significant main or interactions between sex and age group were found in Teacher-rated Anger-Aggression and Anxious Withdrawal, according to children’s sex and age group.

Preliminary correlation analyses found that perceived social motivation (*r* = 0.46, *p* < 0.001), social standing (*r* = 0.40, *p* < 0.001) and sympathy (*r* = 0.49, *p* < 0.001) were positively associated with intended affiliative preferences toward the aggressive peer. Perceived social motivation (*r* = 0.35, *p* < 0.001), social standing (*r* = 0.34, *p* < 0.001) and sympathy (*r* = 0.46, *p* < 0.001) were also positively associated with intended affiliative preferences toward the shy peer. Perceived social motivation (*r* = 0.34, *p* < 0.001), social standing (*r* = 0.32, *p* < 0.001) and sympathy (*r* = 0.42, *p* < 0.001) were positively associated with intended affiliative preferences toward the unsociable peer. Intended affiliative preferences toward all the hypothetical peers were not significantly associated with preschoolers’ perceived intentionality and negative impact. No sociodemographic covariates were identified.

#### 3.2.1. Associations between Preschoolers’ Beliefs, Emotions and Intended Affiliative Preferences toward Peers, Depending on Children’s Teacher-Rated Prosocial Behavior, Sex and Age Group

Table 3 summarizes the results of multiple linear moderated regression analyses to examine the role of children’s teacher-rated prosocial behavior, sex, and age group in the associations between preschoolers’ beliefs (perceived social motivation and social standing), sympathy and intended affiliative preferences toward hypothetical peers. 

##### Aggressive Peers

Preschoolers who perceived aggressive peers as more socially motivated reported higher intended affiliative preferences toward them. The associations between perceived social motivation and intended affiliative preferences toward aggressive peers were moderated by children’s age group and teacher-rated prosocial behavior. When rated as highly prosocial by their teachers, younger children (*t* = 5.34, *p* < 0.001) who perceived aggressive peers as more socially motivated reported higher affiliative preferences toward them than older children (*t* = 3.19, *p* = 0.001). These differences between younger (*t* = 5.34, *p* < 0.001) and older children (*t* = 2.18, *p* = 0.030) were also observed, when children’s teacher-rated prosocial behaviors were moderate. Only younger children who perceived aggressive peers as more socially motivated reported higher affiliative preferences toward them (*t* = 3.01, *p* = 0.003) at low levels of teacher-rated prosocial behaviors.

Preschoolers who perceived aggressive peers as having a higher social standing in the peer group reported higher intended affiliative preferences toward them. The associations between perceived social standing and intended affiliative preferences toward aggressive peers were moderated by children’s teacher-rated prosocial behavior. The magnitude of the associations between perceived social standing and intended affiliative preferences toward aggressive peers was greater at high (*t =* 6.21, *p* < 0.001) and moderate (*t =* 6.66, *p* < 0.001) than at low levels (*t =* 3.03, *p* < 0.001) of prosocial behaviors.

Higher levels of sympathy were associated with higher intended affiliative preferences. No moderation effects of children’s sex, age group and teacher-rated prosocial behavior were identified.

##### Shy Peers

Table 3 also shows that preschoolers who perceived shy peers as more socially motivated reported higher intended affiliative preferences toward them. The associations between perceived social motivation and intended affiliative preferences toward shy peers were moderated by children’s teacher-rated prosocial behavior, age group and sex. When teacher ratings of prosocial behaviors were low, older children (*t =* 4.75, *p* < 0.001) who perceived shy peers as less socially motivated reported higher affiliative preferences toward them than younger children (*t =* 2.97, *p* = 0.003). When rated as moderately (*t =* 4.38, *p* < 0.001) or highly (*t =* 2.26, *p* = 0.025) prosocial by their teachers, only older children who perceived shy peers as more socially motivated reported higher intended affiliative preferences toward them. For boys, the magnitude of the associations between perceived social motivation and intended affiliative preferences was higher when teacher-rated levels of prosocial behaviors were low (*t =* 4.81, *p* < 0.001) and moderate (*t =* 4.39, *p* < 0.001) than high (*t =* 2.14, *p* = 0.033). For girls, the associations between perceived social motivation and intended affiliative preferences toward shy peers were not significant, independent of the level of children’s teacher-rated prosocial behavior.

Preschoolers who perceived higher social standing reported higher intended affiliative preferences. These associations were not moderated by children’s teacher-rated prosocial behavior, sex, or age group. Higher levels of sympathy were associated with higher intended affiliative preferences. These associations were not moderated by children’s teacher-rated prosocial behavior, age, and sex.

##### Unsociable Peers

Table 3 shows that preschoolers who perceived unsociable peers as more socially motivated reported higher affiliative preferences. No moderating effects of children’s teacher-rated prosocial behavior, sex and age group were found.

Older children and preschoolers who perceived unsociable peers as having greater social standing in the peer group reported higher affiliative preferences. The associations between perceived social standing and intended affiliative preferences toward unsociable peers were moderated by children’s teacher-rated prosocial behavior, age group and sex. When rated as highly prosocial by their teachers, older children (*t =* 5.68, *p* < 0.001) who considered that unsociable peers had a greater social standing reported higher affiliative preferences toward them than younger children (*t =* 2.64, *p* = 0.009). These differences between younger (*t =* 1.68, *p* = 0.094) and older children (*t =* 4.94, *p* < 0.001) were also observed at moderate levels of children’s teacher-rated prosocial behaviors. At high levels of children’s teacher-rated prosocial behaviors, boys (*t =* 5.14, *p* < 0.001) who considered that unsociable peers had a greater social standing reported higher affiliative preferences toward them than girls (*t =* 2.84, *p* = 0.005). At moderate levels of teacher-rated prosocial behaviors, only boys who considered that unsociable peers had a greater social standing reported higher affiliative preferences toward them (*t =* 4.57, *p* < 0.001).

Higher levels of sympathy were associated with higher intended affiliative preferences. No moderating effects of children’s age group, sex and teacher-rated prosocial behaviors were found.

#### 3.2.2. Associations between Preschoolers’ Beliefs, Emotions and Intended Affiliative Preferences toward Peers, Depending on Children’s Teacher-Rated Aggression, Sex and Age Group

Table 4 summarizes the findings of multiple linear moderated regression analyses to examine the role of children’s teacher-rated aggression, sex, and age group in the associations between preschoolers’ beliefs (i.e., perceived social motivation and social standing) and affiliative preferences toward hypothetical peers.

##### Aggressive Peers

Children’s teacher-rated aggression didn’t moderate the associations between preschoolers’ beliefs and intended affiliative preferences and between preschoolers’ sympathy and intended affiliative preferences.

##### Shy Peers

Table 4 shows that the positive associations between perceived social motivation and intended affiliative preferences toward shy peers were moderated by children’s teacher-rated aggression, age group and sex. For boys, the magnitude of the associations between perceived social motivation and intended affiliative preferences toward shy peers was stronger, at high (*t =* 5.05, *p* < 0.001) and moderate (*t =* 4.09, *p* < 0.001) than at low (*t =* 2.20, *p* = 0.029) levels of children’s teacher-rated aggression. For girls, the associations between perceived social motivation and intended affiliative preferences toward shy peers were not significant, independent of the level of children’s teacher-rated aggression. For older children, the associations between perceived social motivation and intended affiliative preferences toward shy peers were only significant at high levels of children’s teacher-rated aggression (*t =* 2.78, *p* = 0.006). For younger children, the magnitude of the associations between perceived social motivation and intended affiliative preferences toward shy peers was stronger at high (*t =* 4.87, *p* < 0.001) and moderate (*t =* 3.93, *p* < 0.001) than at low (*t =* 2.22, *p* = 0.02) levels of children’s teacher-rated aggression.

Children’s teacher-rated aggression didn’t moderate the associations between preschoolers’ sympathy and intended affiliative preferences.

##### Unsociable Peers

Children’s teacher-rated aggression didn’t moderate the associations between preschoolers’ beliefs and intended affiliative preferences and between preschoolers’ sympathy and intended affiliative preferences.

#### 3.2.3. Associations between Preschoolers’ Beliefs, Sympathy and Intended Affiliative Preferences toward Peers, Depending on Children’s Teacher-Rated Anxious Withdrawal, Sex and Age Group

Table 5 summarizes the findings of the multiple moderated regression analyses to examine the role of children’s teacher-rated anxious withdrawal, sex, and age group in the associations between preschoolers’ beliefs (i.e., perceived social motivation and social standing) and affiliative preferences toward hypothetical peers.

##### Aggressive Peers

Children’s teacher-rated anxious-withdrawal didn’t moderate the associations between preschoolers’ beliefs and intended affiliative preferences and between preschoolers’ beliefs and sympathy.

##### Shy Peers

No moderating effect of children’s teacher-rated anxious withdrawal was identified in the associations between preschoolers’ beliefs and intended affiliative preferences. However, children’s teacher-rated anxious withdrawal and sex moderated the associations between sympathy and affiliative preferences toward shy peers. For boys, the magnitude of the associations between sympathy and affiliative preferences toward shy peers was stronger at high (*t =* 4.13, *p* < 0.001) and moderate (*t =* 4.63, *p* < 0.001) than at low (*t =* 2.87, *p* = 0.004) levels of children’s teacher-rated anxious withdrawal. For girls, the associations between sympathy and affiliative preferences toward shy peers were not significant, independent of children’s teacher-rated anxious withdrawal.

##### Unsociable Peers

No moderating effect of children’s teacher-rated anxious withdrawal was identified in the associations between preschoolers’ beliefs and intended affiliative preferences and between preschoolers’ sympathy and intended affiliative preferences.

## 4. Discussion

This study aimed to describe Portuguese preschoolers’ beliefs and intended responses toward hypothetical aggressive, shy, and unsociable peers, depending on children’s sex and age group, and to explore the associations between preschoolers’ beliefs and intended responses, depending on children’s social behaviors, sex and age group.

### 4.1. Preschoolers’ Beliefs and Intended Responses toward Aggressive, Shy, Unsociable, and Socially Competent Peers, According to Sex and Age Group

Our findings support our first hypothesis (H1) and prior empirical studies conducted in different cultures [17,20,21,22,23], showing that preschoolers perceived shy and unsociable hypothetical peers as less intentional, less motivated to play and less appreciated in the classroom when compared with socially competent hypothetical peers. In line with our second hypothesis (H2), preschoolers in our sample shared with Argentinian, Canadian, Chinese, Dutch, US, and Italian children [12,17,20,21,22,23] less negative views about the social motivation, social standing and negative impact, greater sympathy and intended affiliative preferences toward shy and unsociable hypothetical peers when compared with aggressive peers. Children’s broad distinctions between social withdrawal and direct aggression support extant evidence, showing that the latter are concurrently and longitudinally associated with negative peer experiences [10].

Notwithstanding these similarities with prior research, our findings only partially support our third hypothesis (H3) about expected differences in children’s perspectives toward shy and unsociable hypothetical peers. As found in Italian and Argentinian samples of younger preschoolers [22,23], participants in our sample reported comparable social standing, negative impact, and affiliative preferences for shyness and unsociability. These findings converge with research, showing that unsociability may be only perceived as problematic by peers during the elementary school years and that negative peer responses begin during middle childhood [11] rather than during early childhood. Contrary to prior studies conducted with younger preschoolers [22,23], our participants were not able to make fine-grained distinctions between shy and unsociable hypothetical peers concerning intentionality and social motivation. In accordance with the bioecological developmental framework [37], macro-time and macro-system factors may have influenced the meaning that preschoolers attributed to unsociable peer behaviors in our sample. Despite the reduction of prophylactic isolation measures during the school year 2021/2022 [49], data collection took place during the third wave of the COVID-19 pandemic crisis, which has continued to be characterized by infection outbreaks in preschool settings. Within this context, the self-imposed isolation of socially withdrawn children in the peer group [2] may have been interpreted in a different way by preschoolers, in terms of their underlying intentionality and motivational substrates. On the other hand, cultural values may have also influenced the findings. In a prior study, Chinese elementary school children were able to distinguish shy and unsociable peers concerning intentionality and social motivation but perceived the behaviors of the latter as less intentional and more socially motivated than Canadian children [20]. Due to social pressures to engage in affiliative and collectivist activities, [20] hypothesized that it may be more conceivable for Chinese children to believe that unsociable peers are still motivated to play and that their behaviors are less intentional. In Portugal, collectivist values related to good manners and respect continue to be perceived as desirable qualities for children [39] and quiet behaviors related to social withdrawal are often encouraged in preschool classrooms [50]. Unsociable peers display a reduced desire for social approach but are capable of socially competent behaviors when approached by peers [5]. Thus, it is possible that younger preschoolers considered that the behaviors of shy and unsociable peers reflect a conformity to social norms, considering them as equally motivated to play and unintentional. However, these explanations need to be tested in future studies.

Our findings diverge from our hypothesis concerning sex differences (H4) in preschoolers’ beliefs and intended responses toward peer behaviors. Only few sex differences were identified in preschoolers’ perceived intentionality about peer behaviors and the identified differences appeared to differ, depending on children’s age group. These findings may be, in part, related to sex differences in the structure, quality and content of peer relationships. In the 3–4 years age group, boys perceived all hypothetical peers as more intentional than girls. Since the early preschool years, boys are typically more prone to play in group and their interactions are more likely to involve dominance and competitive behaviors when compared with girls [51]. In the presence of increased dominant and aggressive behaviors, extant theory [52] and research [53] found that peers’ negative, ambiguous, and benign social behaviors are more likely to be interpreted as intended. This attributional bias may be particularly salient at younger ages, when children’s ability to understand others’ intentions is less sophisticated [18]. In the 5–6 years age group, girls perceived unsociable peers as more intentional than boys. Girls are typically involved in more prolonged interactions with the same peers and are more oriented by prosocial goals than boys [51]. As age advances, girls may become more able to interpret others’ intentionality and recognize the preference of unsociable peers for playing alone, describing them as more intentional than boys. Nevertheless, sex differences need to be interpreted with caution, because preschoolers were only asked to respond to vignettes depicting same-sex peers [22].

Children’s negative perspectives about the social standing and affiliative preferences toward hypothetical aggressive peers were more accentuated in the 5–6 years than in the 3–4 years age group. These findings are partially consistent with expected age differences (H5) that have been previously identified in a sample of Italian younger preschoolers [22]. According to a developmental framework [1], children’s successful engagement in the world of peers is a crucial developmental issue during the preschool years. Preschoolers who are successful at this developmental stage usually display positive affect, are able to modulate arousal effectively, and maintain behavioral organization, so that they are capable to sustain and coordinate interactions with individual peers and to participate in group activities [1]. Deviations from expected adaptive peer engagement among aggressive peers are usually perceived as particularly disruptive for classroom functioning during early childhood [19,54] and may become increasingly salient at the end of the preschool years (5–6 years). Nevertheless, our findings diverge from expected age differences concerning peers’ perceived social motivations [21]. In contrast, preschoolers aged 5–6 years globally perceived hypothetical peers as more motivated to play when compared with preschoolers aged 3–4 years. A closer inspection of descriptive findings evidenced that age differences in perceived social motivation were essentially identified for shy and unsociable hypothetical peers and, to a lesser extent, for socially competent peers. These subtle developmental differences partially converge with previous studies, showing that older children displayed more positive views toward the different subtypes of socially withdrawn children [22] and made more accurate inferences about the social motivations of socially competent peers [21] than younger children. Developmental theory and research also acknowledge that preschoolers engage in more frequent peer interactions and that these interactions become richer, and increasingly complex with age [55]. These developmental changes in the quantitative and qualitative features of peer interactions across preschool years [55] may also explain why older children attributed higher social motivations to hypothetical peers who displayed socially competent or less disruptive social behaviors for the peer group than younger children.

### 4.2. Associations between Preschoolers’ Beliefs, Emotions, and Intended Affiliative Preferences, Depending on Children’s Social Behavior and Sex and Age Group

Contrary to our hypothesis (H6), preschoolers’ inferences about peers’ negative impact in the classroom and intentionality didn’t emerge as significant predictors of affiliative preferences toward problematic peer behaviors as found in prior research [25,26,27]. Methodological features may explain these divergences with prior research. Part of extant studies investigated children’s attributions of intentionality toward hypothetical male peers, displaying clinically significant internalizing (e.g., depression) and externalizing (e.g., hyperactivity and attention deficit) problems [25,26]. These studies were also conducted in samples of kindergarten and primary school children [24], who were found to be more able to distinguish aggressive and socially withdrawn hypothetical peers along a responsibility dimension [12] than younger children. Research drawn on developmental attributional theory suggested that preschoolers’ inferences about peers’ intentionality may be associated with affiliative preferences indirectly, via emotions of sympathy or negative affect [12,28]. Indirect effects of preschoolers’ intentionality in intended affiliative preferences toward peers displaying problematic social behaviors (via children’s positive and negative emotional reactions) need to be tested in future studies.

Notwithstanding these divergences, our findings converge with our hypothesis (H6), showing that preschoolers’ interpretations about peers’ social motivation and social standing and preschoolers’ sympathy consistently predicted intended affiliative preferences toward all hypothetical peers. These findings support theoretical assumptions that children’s intended responses toward peers’ social behaviors are, in part, guided by their emotions and understanding on others’ intentions [12]. However, children’ sex, age group and social behaviors impacted the magnitude of the associations between the aforementioned variables, especially for hypothetical aggressive and shy peers.

For hypothetical aggressive peers, perceived social motivation was a stronger predictor of intended affiliative preferences in the 3–4 years age group. This moderating effect is consistent with the idea that younger preschoolers hold less sophisticated views about peers’ social motivation [18] and may be less nuanced and selective in their intended prosocial initiations with aggressive peers [55]. On the other hand, perceived social standing in the peer group was a stronger predictor of affiliative preferences toward aggressive peers among children rated as more prosocial by teachers. Preschoolers who are more prone to display comforting, helping and cooperating behaviors typically display a more discriminating social sensitivity [18] that may make them more capable to encode and interpret social cues of peers’ likeability in the classroom and may guide their intended affiliative responses. The increased proneness of prosocial children who are better equipped to understand others to engage in defending behaviors against aggression during early childhood (e.g., [56]) may also explain the obtained findings.

For hypothetical shy peers, perceived social motivation was a stronger predictor of intended affiliative preferences in the 5–6 years age group. This moderating effect is consistent with the idea that older preschoolers may hold more advanced social understanding skills [18] which may result in more accurate interpretations of the conflicted approach-avoidance motivations of shy-withdrawn peers. More aggressive and less prossocial boys who perceived shy peers as more socially motivated also reported higher intended affiliative responses toward them. These findings are in line with prior research, suggesting that shy behaviors may carry increased negative costs for boys when compared to girls [2], particularly in the realm of peer interactions [31]. Shy peers may be perceived as deviant from gender norms related to male social dominance and assertion [31,57] and as “easy targets” [2] for more aggressive children. The increased vulnerability of male shy peers in the realm of social interactions [31] may explain why children’s higher levels of sorrow toward shy peers’ negative emotional state (i.e., sympathy) revealed stronger positive associations with intended affiliative responses among boys than among girls in our sample. On the other hand, findings revealed that higher levels of sympathy were more strongly associated with higher levels of intended affiliative preferences toward shy peers among boys, rated as less anxiously withdrawn by teachers. These findings converge with prior research, showing that shy children may be capable of experiencing normative feelings of sorrow toward others’ negative emotional states but may not behaviorally react in accordance with their feelings, due to increased social anxiety and fear of rejection (e.g., [58]).

To a lesser extent, children’s own characteristics were also found to moderate the associations between preschoolers’ beliefs and affiliative preferences toward unsociable hypothetical peers. Perceived social standing was a stronger predictor of affiliative preferences among boys, preschoolers aged 5–6 years and who were rated as more prosocial by teachers. The greater orientation of boys to play in larger groups characterized by more dominant and competitive hierarchies [51] may explain why the anticipated appreciation of unsociable peers in the classroom appeared to be more influential in their intended behavioral responses toward them. Furthermore, these findings support the idea that older and more prossocial children hold a more sophisticated understanding of others’ social cues [18] and use them to guide their intended affiliative preferences.

### 4.3. Limitations and Directions for Future Studies

Notwithstanding its contribution, study limitations need to be acknowledged. First, the findings reflect the perspectives of Portuguese preschoolers and teachers from diverse socioeconomic backgrounds, who were recruited using a convenience sampling method that limits the generalization of the findings. Second, a posteriori power analyses showed that the sample size only allowed us to detect small to medium effects, when testing the associations of each independent variable (i.e., perceived social motivation, social standing or sympathy) on intended affiliative preferences toward each problematic peer behavior (i.e., shy, unsociable, or aggressive), depending on children’s sex, age group, and each teacher-rated social behavior (i.e., prosocial behavior, aggression, or anxious-withdrawal). This allowed us to clarify the moderating role of each teacher-rated social behavior, considering its addictive effects with children’s sex and age group. However, the sample size didn’t allow us to test all the independent variables, moderators, and interaction terms together in the same model. Third, the structured interview used in the present study was validated in several cultures and allowed us to explore children’s interpretations, emotions, and affiliative responses toward hypothetical peers. Nevertheless, we cannot ignore that children’s interpretations, emotions, and affiliative responses toward aggressive, shy, unsociable, and socially competent peers were assessed with a reduced number of items and may be different in naturalistic preschool settings. Fourth, data collection took place during the third wave of the COVID-19 pandemic crisis. The timing of data collection may have influenced preschoolers’ interpretations toward hypothetical peer behaviors, but also teacher ratings of children’s social behaviors. Lastly, the cross-sectional design of the present study didn’t allow to infer causal relations between the study variables.

Future cross-cultural and longitudinal studies based on a multi-method and multi-informant approach (e.g., children’s interviews, preschool observations, sociometric measures, teacher, and parent sympathy ratings) may contribute to examine the cultural commonalities and differences and the developmental changes in preschoolers’ interpretations, emotions, and affiliative responses toward hypothetical and real peers. Future studies need to examine the associations between preschoolers’ beliefs and emotions considered together and intended affiliative preferences toward peer problematic behaviors. Similarly, moderated moderation models may be useful to clarify the complex interactions of children’s social behaviors (i.e., prosocial behavior, aggression, and anxious withdrawal) considered together with children’s sex, age group and classroom-level factors (e.g., mean classroom level of social behaviors or classroom climate) in the previously described associations. The indirect associations between preschoolers’ interpretations and affiliative responses, via positive and negative emotional reactions, may need also to be examined to shed light on the processes and mechanisms that need to be targeted in future intervention programs.

## 5. Conclusions

Overall, our findings that preschoolers’ perspectives toward shy and unsociable peers were less negative than toward aggressive peers. However, participants in our sample were not fully aware of the different intentionality and social motivations of shy and unsociable peers. Higher levels of perceived social standing, social motivation and sympathy predicted higher affiliative preferences toward shy, unsociable, and aggressive peers. Nevertheless, the magnitude of the associations between preschoolers’ beliefs, emotions and intended affiliative preferences differed, depending on children’s sex, age group and prosocial behavior, especially toward aggressive and shy peer behaviors.

These findings appear to support the need to design evidence-based intervention programs targeted at young children to be implemented in preschool settings that may promote adaptive beliefs and responses toward aggressive, shy, and unsociable peers and enhance their successful inclusion in the classroom. These interventions need to be developmentally tailored, as well as to consider sex differences in peer relationships and children’s individual differences in social behaviors in the classroom.

## Figures and Tables

**Table 1:** Preschoolers’ Beliefs (Perceived Intentionality, Social Motivation, Social Standing, and Negative Impact), Sympathy and Intended Responses (Affiliative Preferences) toward Aggressive, Shy, Unsociable and Socially Competent Peer Behaviors, according to Children’s Sex and Age Group.

	Aggressive Peer Behaviors					Shy Peer Behaviors					Unsociable Peer Behaviors					Socially Competent Peer Behaviors				
	Boys	Girls	3–4 y	5–6 y	Total	Boys	Girls	3–4 y	5–6 y	Total	Boys	Girls	3–4 y	5–6 y	Total	Boys	Girls	3–4 y	5–6 y	Total
	M (SD)	M (SD)	M (SD)	M (SD)	M (SD)	M (SD)	M (SD)	M (SD)	M (SD)	M (SD)	M (SD)	M (SD)	M (SD)	M (SD)	M (SD)	M (SD)	M (SD)	M (SD)	M (SD)	M (SD)
INT	1.93 (0.81)	1.81 (0.77)	1.92 (0.78)	1.86 (0.81)	1.89 (0.79)	2.03 (0.68)	1.70 (0.71)	1.91 (0.73)	1.66 (0.74)	1.79 (0.74)	1.97 (0.76)	1.95 (0.75)	2.06 (0.78)	1.84 (0.72)	1.96 (0.76)	2.34 (0.73)	2.21 (0.78)	2.26 (0.74)	2.32 (0.77)	2.29 (0.75)
Mixed ANOVA statistics. Type of Behavior: *F* = 16.66 ***, η^2^p = 0.251. Sex: *F* = 1.44, η^2^p = 0.009. Age Group: *F =* 0.61, η^2^p = 0.004. Age Group × Sex: *F =* 11.12 ***, η^2^p = 0.069. Type of Behavior × Sex: *F* = 0.29, η^2^p =.002. Type of Behavior × Age Group: *F =* 1.52, η^2^p = 0.010.																				
SM	1.96 (0.85)	1.93 (0.87)	1.96 (0.82)	1.93 (0.89)	1.95 (0.86)	2.13 (0.87)	2.36 (0.76)	2.09 (0.83)	2.34 (0.83)	2.22 (0.83)	2.17 (0.82)	2.20 (0.83)	2.04 (0.80)	2.33 (0.82)	2.19 (0.82)	2.49 (0.73)	2.59 (0.67)	2.47 (0.74)	2.59 (0.67)	2.53 (0.71)
Mixed ANOVA statistics. Type of Behavior: *F =* 14.87 ***, η^2^p = 0.029. Sex: *F* = 1.16, η^2^p =.008. Age Group: *F* = 4.15 *, η^2^p = 0.027. Age Group × Sex: *F* = 0.03, η^2^p = 0.000. Type of Behavior × Sex: *F* = 0.73, η^2^p = 0.005. Type of Behavior × Age Group: *F =* 0.92, η^2^p = 0.006.																				
SS	1.80 (0.87)	1.89 (0.86)	1.93 (0.82)	1.74 (0.90)	1.84 (0.86)	2.32 (0.82)	2.46 (0.73)	2.28 (0.84)	2.47 (0.73)	2.44 (0.74)	2.43 (0.73)	2.44 (0.76)	2.49 (0.68)	2.38 (0.79)	2.44 (0.74)	2.49 (0.72)	2.65 (0.58)	2.46 (0.68)	2.64 (0.65)	2.55 (0.67)
Mixed ANOVA statistics. Type of Behavior: *F* = 29.77 ***, η^2^p = 0.17. Sex: *F* = 1.46, η^2^p = 0.010. Age Group: *F =* 0.02, η^2^p = 0.000. Age Group × Sex: *F* = 0.17, η^2^p = 0.001. Type of Behavior × Sex: *F* = 0.37, η^2^p = 0.003. Type of Behavior × Age Group: *F* = 2.75 *, η^2^p = 0.019																				
NI	2.15 (0.85)	2.17 (0.87)	2.13 (0.89)	2.18 (0.82)	2.15 (0.85)	1.78 (0.86)	1.75 (0.80)	1.82 (0.88)	1.71 (0.79)	1.77 (0.83)	1.79 (0.79)	1.93 (0.86)	2.00 (0.82)	1.68 (0.80)	1.85 (0.82)	1.70 (0.83)	1.60 (0.85)	1.70 (0.88)	1.62 (0.79)	1.66 (0.74)
Mixed ANOVA statistics. Type of Behavior: *F =* 14.94 ***, η^2^p = 0.093. Sex: *F* = 0.01, η^2^p = 0.000. Age Group: *F* = 0.78, η^2^p = 0.005. Age Group × Sex: *F* = 1.64, η^2^p = 0.011. Type of Behavior × Sex: *F* = 0.74, η^2^p = 0.005. Type of Behavior × Age Group: *F* = 1.97, η^2^p = 0.013.																				
SY	1.92 (0.84)	1.76 (0.82)	1.90 (0.81)	1.80 (0.85)	1.85 (0.83)	2.18 (0.82)	2.34 (0.79)	2.12 (0.79)	2.37 (0.81)	2.24 (0.81)	2.28 (0.79)	2.42 (0.81)	2.29 (0.76)	2.27 (0.82)	2.34 (0.79)	2.28 (0.85)	2.19 (0.86)	2.20 (0.82)	2.29 (0.89)	2.24 (0.85)
	Mixed ANOVA statistics. Type of Behavior: *F =* 14.69 ***, η^2^p = 0.090. Sex: *F* = 0.03, η^2^p = 0.000. Age Group: *F* = 0.90, η^2^p = 0.006. Age Group × Sex: *F* = 0.53, η^2^p = 0.003. Type of Behavior × Sex: *F* = 1.99, η^2^p = 0.012. Type of Behavior × Age Group: *F* = 1.93, η^2^p = 0.012.																			
AP	1.88 (0.68)	1.75 (0.63)	1.91 (0.65)	1.75 (0.66)	1.83 (0.66)	2.45 (0.67)	2.58 (0.57)	2.47 (0.65)	2.54 (0.61)	2.50 (0.63)	2.48 (0.63)	2.50 (0.59)	2.41 (0.63)	2.57 (0.60)	2.49 (0.62)	2.51 (0.55)	2.62 (0.54)	2.49 (0.55)	2.55 (0.57)	2.55 (0.55)
Mixed ANOVA statistics. Type of Behavior: *F =* 65.52 ***, η^2^p = 0.301. Sex: *F =* 0.26, η^2^p = 0.002. Age Group: *F =* 0.57, η^2^p = 0.004. Age Group × Sex: 0.01, η^2^p = 0.000. Type of Behavior × Sex: *F* = 1.70, η^2^p = 0.012. Type of Behavior × Age Group: *F* = 2.89 *, η^2^p = 0.019.																				

INT refers to Perceived Intentionality. SM refers to Perceived Social Motivation. SS refers to Perceived Social Standing. NI refers to Perceived Negative Impact. SY refers to Perceived Sympathy. AP refers to Perceived Affiliative Preference. *** *p* < 0.001. * *p* < 0.05.

**Table 2:** Descriptive Statistics (Means and Standard Deviations) of Teacher-Rated Prosocial Behavior, depending on Children’s Sex and Age Group.

	Boys	Girls	3–4 y	5–6 y	Total
	M (SD)	M (SD)	M (SD)	M (SD)	M (SD)
Prosocial Behavior	3.90 (0.88)	4.30 (0.68)	3.87 (0.84)	4.26 (0.78)	4.06 (0.83)
Model statistics: Sex: *F* = 9.77, *p* = 0.002, η^2^p = 0.060. Age Group: *F* = 7.15, *p* = 0.008, η^2^p = 0.045. Sex × Age Group: *F* = 3.06, *p* = 0.083, η^2^p = 0.020					
Anger-Aggression	2.21 (0.88)	2.02 (0.61)	2.20 (0.91)	2.06 (0.63)	2.13 (0.79)
Model statistics: Sex: *F* = 2.10, *p* = 0.150, η^2^p = 0.014. Age Group: *F* = 0.73, *p* = 0.393, η^2^p = 0.005. Sex × Age Group: *F* = 1.08, *p* = 0.300, η^2^p = 0.007					
Anxious-Withdrawal	2.11 (0.62)	2.12 (0.52)	2.08 (0.55)	2.16 (0.62)	2.12 (0.59)
Model statistics: Sex: *F* = 0.00, *p* = 0.947, η^2^p = 0.000. Age Group: *F* = 0.54, *p* = 0.466, η^2^p = 0.004. Sex × Age Group: *F* = 0.04, *p* = 0.852, η^2^p = 0.000.					

**Table 3:** Associations between Preschoolers’ Beliefs and Affiliative Preferences toward Peer Behaviors, depending on Children’s Teacher-Rated Prosocial Behavior, Sex and Age Group.

	Affiliative Preferences					
	Aggressive Peer		Shy Peer		Unsociable Peer	
Step 1.	β	*t*	β	*t*	β	*t*
SM	0.40	5.48 ***	0.32	4.15 ***	0.26	3.28 **
PB	−0.07	−0.88	0.02	0.33	−0.03	−0.40
Sex	0.07	0.96	−0.05	−0.57	0.00	0.04
Age Group	0.10	1.37	0.01	0.13	−0.12	−1.47
Step 2.	Δ*F* = 2.54 ^+^, Δ*R*^2^ = 0.04		Δ*F* = 4.09 **, Δ*R*^2^ = 0.07		Δ*F* = 0.10, Δ*R*^2^ = 0.00	
SM × PB	0.13	1.71 ^+^	−0.18	−2.35 *	-	-
SM × Sex	0.04	0.32	0.21	1.63 ^+^	-	-
SM × Age Group	0.25	2.44 *	−0.22	−2.06 *	-	-
*Model statistics*	*F =* 6.42 ***, *R*^2^ = 0.23		*F* = 4.76 ***, *R*^2^ = 0.18		*F =* 2.08 *, *R*^2^ = 0.09	
Step 1.	β	*t*	β	*t*	β	*t*
SS	0.46	6.44 ***	0.35	4.57 ***	0.33	4.35 ***
PB	−0.06	−0.84	0.07	0.91	−0.02	−0.25
Sex	0.09	1.17	−0.06	−0.72	−0.01	−0.14
Age Group	0.06	0.77	−0.01	−0.07	−0.17	−2.07 *
Step 2.	Δ*F* = 2.24 ^+^, Δ*R*^2^ = 0.03		Δ*F* = 0.21, Δ*R*^2^ = 0.00		Δ*F* = 5.03 **, Δ*R*^2^ = 0.08	
SS × PB	0.16	2.14 *	-	-	0.20	2.50*
SS × Sex	0.16	1.54	-	-	0.20	1.69 ^+^
SS × Age Group	0.00	0.04			−0.20	−1.95 ^+^
*Model statistics*	*F =* 7.96 ***, *R*^2^ = 0.27		*F* = 3.44 **, *R*^2^ = 0.14		*F =* 5.58 ***, *R*^2^ = 0.21	
Step 1.	β	*t*	β	*t*	β	*t*
SY	0.49	6.86 ***	0.46	6.26 ***	0.40 *	5.41 ***
PB	−0.01	−0.14	0.09	1.14	−0.01	−0.10
Sex	0.03	0.38	−0.04	0.50	0.02	0.38
Age Group	0.09	1.17	0.04	0.54	−0.14	−1.87 ^+^
Step 2.	Δ*F* = 0.67, Δ*R*^2^ = 0.01		Δ*F* = 1.72, Δ*R*^2^ = 0.03		Δ*F* = 0.07, Δ*R*^2^ = 0.00	
SY × PB	-	-	-	-	-	-
SY × Sex	-	-	-	-	-	-
SY × Age Group	-	-	-	-	-	-
*Model statistics*	*F* = 7.82 ***, *R*^2^ = 0.27		*F* = 6.87 ***, *R*^2^ = 0.24		*F* = 4.77 ***, *R*^2^ = 0.18	

*Notes.* Multiple linear moderated regression analyses. SM refers to perceived Social Motivation. PB refers to Teacher-rated Prosocial Behavior. SS refers to perceived Social Standing. SY refers to Sympathy. Sex: dummy-coded as 0 (female), and 1 (male). Age group: dummy-coded as 0 (5–6 years), and 1 (3–4 years). Interaction terms marked with a dash are not reported, because their introduction didn’t significantly improve the explained variance of the model. ^+^ *p* < 0.10. * *p* < 0.05. ** *p* < 0.01. *** *p* < 0.001.

**Table 4:** Associations between Preschoolers’ Beliefs and Affiliative Preferences toward Peer Behaviors, depending on Children’s Teacher-Rated Aggression, Sex and Age Group.

	Affiliative Preferences					
	Aggressive Peer		Shy Peer		Unsociable Peer	
Step 1	β	*t*	β	*t*	β	*t*
SM	0.40	5.52 ***	0.32	4.04 ***	0.25	3.20 ***
AG	0.01	0.12	−0.06	0.78	−0.05	−0.65
Sex	0.09	1.16	−0.05	0.58	0.02	0.21
Age Group	0.12	1.62	0.01	0.08	−0.11	1.35
Step 2.	Δ*F* = 1.74, Δ*R*^2^ = 0.03		Δ*F* = 4.19 ***, Δ*R*^2^ = 0.07		Δ*F* = 0.11, Δ*R*^2^ = 0.00	
SM × AG	-	-	0.19	2.39 *	-	-
SM × Sex	-	-	0.23	1.84 ^+^	-	-
SM × Age Group	-	-	−0.21	−1.92 ^+^	-	-
Model statistics	*F =* 5.87 ***, *R*^2^ = 0.22		*F =* 4.89 ***, *R*^2^ = 0.19		*F =* 2.13 *, *R*^2^ = 0.09	
Step 1.	β	*t*	β	*t*	β	*t*
SS	0.46	6.47 ***	0.34	4.50 ***	0.33	4.39 ***
AG	0.00	0.01	−0.10	−1.31	−0.08	−1.06
Sex	0.10	1.43	−0.06	−0.78	0.00	0.06
Age Group	0.08	1.06	−0.02	−0.21	−0.15	−1.97 ^+^
Step 2.	Δ*F* = 1.28, Δ*R*^2^ = 0.02		Δ*F* = 0.22, Δ*R*^2^ = 0.00		Δ*F* = 3.39, Δ*R*^2^ = 0.05	
SS × AG	-	-	-	-	−0.06	−0.82
SS × Sex	-	-	-	-	0.21	1.78 ^+^
SS × Age Group	-	-	-	-	−0.25	−2.51 *
Model statistics	*F =* 7.27 ***, *R*^2^ = 0.26		*F =* 3.59 **, *R*^2^ = 0.15		*F =* 4.91 ***, *R*^2^ = 0.19	
Step 1.	β	*t*	β	*t*	β	***t***
SY	0.49	6.96 ***	0.45	6.18 ***	0.40	5.42 ***
AG	−0.02	−0.21	−0.09	1.26	−0.06	−0.85
Sex	0.03	0.44	−0.05	−0.63	0.04	0.53
Age Group	0.09	1.25	0.03	0.35	−0.13	−1.83 ^+^
Step 2.	Δ*F* = 0.96, Δ*R*^2^ = 0.01		Δ*F* = 1.70, Δ*R*^2^ = 0.03		Δ*F* = 0.09, Δ*R*^2^ = 0.00	
SY × AG	-	-	-	-	-	-
SY × Sex	-	-	-	-	-	-
SY × Age Group	-	-	-	-	-	-
Model statistics	*F =* 7.99 ***, *R*^2^ = 0.27		*F =* 6.91 ***, *R*^2^ = 0.25		*F =* 4.90 ***, *R*^2^ = 0.19	

*Notes.* Multiple linear moderated regression analyses. SM refers to perceived Social Motivation. AG refers to Teacher-rated Aggression. SS refers to perceived Social Standing. SY refers to Sympathy. Sex: dummy-coded as 0 (female) and 1 (male). Age group: dummy-coded as 0 (5–6 years) and 1 (3–4 years). Interaction terms marked with a dash are not reported, because their introduction didn’t significantly improve the explained variance of the model. ^+^ *p* < 0.10. * *p* < 0.05. ** *p* < 0.01. *** *p* < 0.001.

**Table 5:** Associations between Preschoolers’ Beliefs and Affiliative Preferences toward Peer Behaviors, depending on Children’s Teacher-Rated Anxious-Withdrawal, Sex and Age Group.

	Affiliative Preferences					
	Aggressive Peer		Shy Peer		Unsociable Peer	
Step 1.	β	*t*	β	*t*	β	*t*
SM	0.41	5.58 ***	0.33	4.18 ***	0.26	3.27 **
AW	−0.06	−0.76	−0.01	0.07	−0.03	−0.39
Sex	0.09	1.16	−0.05	−0.67	0.01	0.12
Age Group	0.12	1.59	−0.00	−0.04	−0.11	−1.44
Step 2.	Δ*F* = 1.33, Δ*R*^2^ = 0.02		Δ*F* = 2.47^+^, Δ*R*^2^ = 0.04		Δ*F* = 0.10, Δ*R*^2^ = 0.00	
SM × AW	-	-	0–07	0.89	-	-
SM × Sex	-	-	0.26	2.02 *	-	-
SM × Age Group			−0.17	1.57	-	-
Model statistics	*F =* 5.74 ***, *R*^2^ = 0.21		*F =* 3.96 ***, *R*^2^ = 0.16		*F =* 2.08 *, *R*^2^ = 0.09	
Step 1.	β	*t*	β	*t*	β	*t*
SS	0.48	6.63 ***	0.35	4.51 ***	0.33	4.37 ***
AW	−0.09	−1.29	−0.02	−0.31	−0.03	−0.40
Sex	0.10	1.41	−0.07	−0.97	−0.01	−0.09
Age Group	0.07	0.96	−0.03	−0.33	−0.16	−2.10 *
Step 2.	Δ*F* = 0.91, Δ*R*^2^ = 0.01		Δ*F* = 0.23, Δ*R*^2^ = 0.00		Δ*F* = 3.01 *, Δ*R*^2^ = 0.05	
SS × AW	-	-	-	-	−0.06	−0.70
SS × Sex	-	-	-	-	0.18	1.51
SS × Age Group	-	-	-	-	−0.26	−2.59 *
Model statistics	*F =* 7.38 ***, *R*^2^ = 0.26		*F =* 3.33 **, *R*^2^ = 0.14		*F =* 4.60 ***, *R*^2^ = 0.18	
Step 1.	**β**	***t***	**β**	***t***	**β**	***t***
SY	0.49	6.97 ***	0.45	6.16 ***	0.40	5.43 ***
AW	−0.04	−0.54	−0.01	0.18	0.03	0.40
Sex	0.03	0.39	−0.06	−0.78	0.03	0.43
Age Group	0.09	1.21	0.02	0.24	−0.14	1.88 ^+^
Step 2.	Δ*F* = 0.82, Δ*R*^2^ = 0.01		Δ*F* = 2.54 *, Δ*R*^2^ = 0.04		Δ*F* = 0.68, Δ*R*^2^ = 0.01	
SY × AW	-	-	−0.13	1.81^+^	-	-
SY × Sex	-	-	0.23	2.01*	-	-
SY × Age Group	-	-	−0.07	−0.68	-	-
Model statistics	*F =* 7.96 ***, *R*^2^ = 0.26		*F =* 7.19 ***, *R*^2^ = 0.25		*F =* 5.11 ***, *R*^2^ = 0.19	

Notes. Multiple linear moderated regression. SM refers to perceived Social Motivation. AW refers to Teacher-rated Anxious Withdrawal. SS refers to perceived Social Standing. Sex: dummy-coded as 0—female, 1—male. Age group: dummy-coded as 0—5–6 years, 1—3–4 years. Interaction terms marked with a dash were not reported, because their introduction didn’t significantly improve the explained variance of the model. ^+^ *p* < 0.10. * *p* < 0.05. ** *p* < 0.01. *** *p* < 0.001.

## Data Availability

The raw data supporting the conclusions of this article will be made available by the authors, under request.

## References

[1] Sroufe L.A., Egeland B., Carlson E.A., Collins W.A., Laursen B. (1999). One social world: The integrated development of parent-child and peer relationships. Relationships as Developmental Contexts.

[2] Rubin K.H., Coplan R.J., Bowker J.C. (2009). Social withdrawal in childhood. Annu. Rev. Psychol..

[3] Coplan R.J., Baldwin D., Wood K.R., Schmidt L.A., Poole K.L. (2020). Shy but getting by: Protective factors in the links between childhood shyness and socio-emotional functioning. Adaptive Shyness: Multiple Perspectives on Behavior and Development.

[4] Coplan R.J., Prakash K., O’Neil K., Armer M. (2004). Do you ”want” to play? Distinguishing between conflicted shyness and social disinterest in early childhood. Dev. Psychol..

[5] Coplan R.J., Weeks M. (2010). Unsociability in middle childhood: Conceptualization, assessment, and associations with socioemotional functioning. Merrill-Palmer Q..

[6] Rubin K.H., Chronis-Tuscano A. (2021). Perspectives on social withdrawal in childhood: Past, present, and prospects. Child Dev. Perspect..

[7] Ostrov J.M., Blakely-McClure S.J., Perry K.J., Kampar-DeMarco K.E., Coyne S.M., Ostrov J.M. (2018). Definitions—The form and function of relational aggression. The Development of Relational Aggression.

[8] Sette S., Baumgartner E., Schneider B.H. (2014). Shyness, child–teacher relationships, and socio-emotional adjustment in a sample of Italian preschool-aged children. Infant Child Dev..

[9] Zhang L., Eggum-Wilkens N.D., Eisenberg N., Spinrad T.L. (2017). Children’s shyness, peer acceptance, and academic achievement in the early school years. Merrill-Palmer Q..

[10] Yue X., Zhang Q. (2023). The association between peer rejection and aggression types: A meta-analysis. Child Abus. Negl..

[11] Coplan R.J., Ooi L.L., Baldwin D. (2019). Does it matter when we want to be alone? Exploring developmental timing effects in the implications of unsociability. New Ideas Psychol..

[12] Graham S., Hoehn S. (1995). Children’s understanding of aggression and withdrawal as social stigmas: An attributional analysis. Child Dev..

[13] Dunn J., Cutting A.L., Fisher N. (2002). Old friends, new friends: Predictors of children’s perspective on their friends at school. Child Dev..

[14] Wellman H.M. (2014). Making Minds: How Theory of Mind Develops.

[15] Bukowski W.M. (1990). Age differences in children’s memory of information about aggressive, socially withdrawn, and prosocial boys and girls. Child Dev..

[16] Younger A.J., Schwartzman A.E., Ledingham J.E. (1996). Age-related differences in children’s perceptions of social deviance: Changes in behavior or in perspective?. Dev. Psychol..

[17] Coplan R.J., Girardi A., Findlay L.C., Frohlick S.L. (2007). Understanding solitude: Young children’s attitudes and responses toward hypothetical socially withdrawn peers. Soc. Dev..

[18] Findlay L.C., Girardi A., Coplan R.J. (2006). Links between empathy, social behavior, and social understanding in early childhood. Early Child. Res. Q..

[19] Goossens F.A., Bokhorst K., Bruinsma C., van Boxtel H.W. (2002). Judgments of aggressive, withdrawn, and prosocial behavior: Perceived control, anger, pity, and sympathy in young Dutch children. J. Sch. Psychol..

[20] Coplan R.J., Zheng S., Weeks M., Chen X. (2012). Young children’s perceptions of social withdrawal in China and Canada. Early Child Dev. Care.

[21] Ding X., Coplan R.J., Sang B., Liu J., Pan T., Cheng C. (2015). Young Chinese children’s beliefs about the implications of subtypes of social withdrawal: A first look at social avoidance. Br. J. Dev. Psychol..

[22] Zava F., Watanabe L.K., Sette S., Baumgartner E., Laghi F., Coplan R.J. (2020). Young children’s perceptions and beliefs about hypothetical shy, unsociable, and socially avoidant peers at school. Soc. Dev..

[23] Castillo K.N., Greco C., Korzeniowski C., Ison M.S., Coplan R.J. (2022). Young Argentine children’s attributions about hypothetical socially withdrawn peers. J. Genet. Psychol..

[24] Hennessy E., Swords L., Heary C. (2008). Children’s understanding of psychological problems displayed by their peers: A review of the literature. Child Care Health Dev..

[25] Peterson L., Mullins L.L., Ridley-Johnson R. (1985). Childhood depression: Peer reactions to depression and life stress. J. Abnorm. Child Psychol..

[26] Sigelman C.K., Begley N.L. (1987). The early development of reactions to peers with controllable and uncontrollable problems. J. Pediatr. Psychol..

[27] Swords L., Heary C., Hennessy E. (2011). Factors associated with acceptance of peers with mental health problems in childhood and adolescence. J. Child Psychol. Psychiatry.

[28] Juvonen J. (1991). Deviance, perceived responsibility, and negative peer reactions. Dev. Psychol..

[29] Boxer P., Tisak M.S. (2005). Children’s beliefs about the continuity of aggression. Aggress. Behav..

[30] Giles J.W., Heyman G.D. (2003). Preschoolers’ beliefs about the stability of antisocial behavior: Implications for navigating social challenges. Soc. Dev..

[31] Doey L., Coplan R.J., Kingsbury M. (2014). Bashful boys, and coy girls: A review of gender differences in childhood shyness. Sex Roles.

[32] Fernandes M., Santos A.J., Antunes M., Fernandes C., Monteiro L., Vaughn B.E., Veríssimo M. (2020). Convergent and discriminant validities of SCBE-30 questionnaire using correlated trait–correlated method Minus One. Front. Psychol..

[33] Echeverría A.V., Rocha T., Leite J.C., Teixeira P., Cruz O. (2016). Portuguese validation of the social competence and behavior evaluation scale (SCBE-30). Psicol. Reflex. Crit..

[34] LaFreniere P.J., Dumas J.E. (1996). Social competence and behavior evaluation in children ages 3 to 6 years: The short form (SCBE-30). Psychol. Assess..

[35] LaFreniere P., Masataka N., Butovskaya M., Chen Q., Auxiliadora Dessen M., Atwanger K., Schreiner S., Montirosso R., Frigerio A. (2002). Cross-cultural analysis of social competence and behavior problems in preschoolers. Early Educ. Dev..

[36] Sette S., Baumgartner E., MacKinnon D.P. (2015). Assessing social competence and behavior problems in a sample of Italian preschoolers using the social competence and behavior evaluation scale. Early Educ Dev..

[37] Bronfenbrenner U., Morris P.A., Lerner R.M., Damon W. (2006). The bioecological model of human development. Handbook of Child Psychology: Theoretical Models of Human Development.

[38] Chen X., Lee J., Chen L., Bukowski W.B., Laursen B., Rubin K.H. (2018). Culture and peer relationships. Handbook of Peer Interactions, Relationships, and Groups.

[39] Ramos A., Magalhães P.C. (2022). European Values Study: Relatório do Estudo dos Valores Europeus, 2017–2019.

[40] Santos A.J., Monteiro L., Sousa T., Fernandes C., Torres N., Vaughn B. (2015). Low social engagement: Implications for children psychosocial adjustment in the preschool context. Psicol. Reflex. Crit..

[41] Santos A.J., Daniel J.R., Antunes M., Coppola G., Trudel M., Vaughn B.E. (2020). Changes in preschool children’s social engagement positively predict changes in social competence: A three-year longitudinal study of Portuguese children. Soc. Dev..

[42] Vaughn B.E., Santos A.J., Monteiro L., Shin N., Daniel J.R., Krzysik L., Pinto A. (2016). Social engagement and adaptive functioning during early childhood: Identifying and distinguishing among subgroups differing with regard to social engagement. Dev. Psychol..

[43] Coelho L., Torres N., Fernandes C., Santos A.J. (2017). Quality of play, social acceptance, and reciprocal friendship in preschool children. Eur. Early Child. Educ. Res. J..

[44] Coplan R.J., Ooi L.L., Rose-Krasnor L., Nocita G. (2014). ‘I Want to Play Alone’: Assessment and correlates of self-reported preference for solitary play in young children. Infant Child Dev..

[45] Aiken L.S., West S.G. (1991). Multiple Regression: Testing and Interpreting Interactions.

[46] Hayes A.F. (2018). Introduction to Mediation, Moderation, and Conditional Process Analysis: A Regression-Based Approach.

[47] Faul F., Erdfelder E., Buchner A., Lang A.-G. (2009). Statistical power analyses using G∗Power 3.1: Tests for correlation and regression analyses. Behav. Res. Methods.

[48] Faul F., Erdfelder E., Lang A.G. (2007). G-Power3: A flexible statistical power analysis program for the social, behavioural, and biomedical sciences. Behav. Res. Methods.

[49] Direção Geral de Saúde (2021). Referencial Escolas—Controlo da Transmissão de COVID-19 em Contexto Escolar.

[50] Guedes M., Coelho L., Santos A.J., Veríssimo M. (2019). Perceptions of Portuguese psychologists about behavioural inhibition/social withdrawal and their related intervention needs during early childhood. Psicologia.

[51] Rose A.J., Smith R.L., Bukowski W.B., Laursen B., Rubin K.H. (2018). Gender and peer relationships. Handbook of Peer Interactions, Relationships, and Groups.

[52] Dodge K.A. (1980). Social cognition and children’s aggressive behavior. Child Dev..

[53] Martinelli A., Ackermann K., Bernhard A., Freitag C.M., Schwenck C. (2018). Hostile attribution bias and aggression in children and adolescents: A systematic literature review on the influence of aggression subtype and gender. Aggress. Violent Behav..

[54] Coplan R.J., Bullock A., Archbell K.A., Bosacki S. (2015). Preschool teachers’ attitudes, beliefs, and emotional reactions to young children’s peer group behaviors. Early Child. Res. Q..

[55] Coplan R.J., Arbeau K.A., Rubin K.H., Bukowski W.M., Laursen B. (2009). Peer interactions and play in early childhood. Handbook of Peer Interactions, Relationships, and Groups.

[56] Swit C.S., Blakely-McClure S.J., Kamper-DeMarco K.K. (2023). Preventing bullying in preschool-age children: Predictors of defending behaviour. Int. J. Bullying Prev..

[57] Sette S., Colasante T., Zava F., Baumgartner E., Malti T. (2018). Preschoolers’ anticipation of sadness for excluded peers, sympathy, and prosocial behavior. J. Genet. Psychol..

[58] Zava F., Sette S., Baumgartner E., Coplan R.J. (2021). Shyness and empathy in early childhood: Examining links between feelings of empathy and empathetic behaviours. Br. J. Dev. Psychol..

